# Study of SOX combined with intraperitoneal high‐dose paclitaxel in gastric cancer with synchronous peritoneal metastasis: A phase II single‐arm clinical trial

**DOI:** 10.1002/cam4.5277

**Published:** 2022-09-26

**Authors:** Li Tu, Weihan Zhang, Lu Ni, Zihan Xu, Kun Yang, Hongfeng Gou, Qing Zhu, Ming Liu, Yu Yang, Jiankun Hu, Meng Qiu

**Affiliations:** ^1^ Department of Medical Oncology, Cancer Center and State Key Laboratory of Biotherapy West China Hospital, Sichuan University Chengdu China; ^2^ Department of Gastrointestinal Surgery and Laboratory of Gastric Cancer, State Key Laboratory of Biotherapy West China Hospital, Sichuan University, and Collaborative Innovation Center for Biotherapy Chengdu China

**Keywords:** chemotherapy, gastric cancer, oxaliplatin, paclitaxel, S‐1

## Abstract

**Background:**

Intraperitoneal paclitaxel is proved to be efficient for peritoneal metastasis of gastric cancer. It remains uncertain the efficacy and safety of the triplets regimen which combined intraperitoneal high‐dose paclitaxel with systemic SOX in gastric cancer patients with peritoneal metastasis. This study aimed to evaluate the efficacy and safety of intraperitoneal administration of high‐dose paclitaxel, intravenous oxaliplatin and S‐1 in patients with peritoneal metastatic gastric cancer.

**Methods:**

This single‐center, prospective, single‐arm phase II study was conducted between January 2017 and May 2019 in West China Hospital, Sichuan University. Patients diagnosed with primary gastric cancer by histopathology and confirmed synchronous peritoneal metastasis were enrolled. This study aimed to evaluate efficacy and safety of intraperitoneal administration of high‐dose paclitaxel (80 mg/m^2^, d1), intravenous oxaliplatin (100 mg/m^2^, d1), and S‐1 (80 mg/m^2^, d1‐14) of patients. The primary endpoint was 1‐year overall survival rate, and the second endpoints were progression‐free survival (PFS), overall survival (OS), overall response rate (ORR), disease control rate (DCR) and adverse events.

**Results:**

In this single‐arm phase II clinical trial, 49 patients received SOX combined intraperitoneal high‐dose paclitaxel treatment. One‐year survival rate was 81.6% (95% CI, 68.6–90.0%). Median PFS and OS were 6.50 months (95% CI, 2.89–10.11) and 16.9 months (95% CI, 13.58 to 20.22), respectively; ORR was 55.3% (95% CI, 41.3–68.6) and DCR was 76.6% (95% CI, 62.8–86.4). Thirteen patients underwent second laparoscopic detection, but only nine ultimately underwent radical gastrectomy. Subgroup analysis showed that sPCI ≤12 was a good index for a favorable prognosis. The most frequent grade 3/4 toxicities were neutropenia (40.8%), anemia (22.4%), leukopenia (18.4%), nausea (14.3%), and vomiting (12.2%). None of the patients had any intraperitoneal catheter‐related complications.

**Conclusions:**

Intraperitoneal high‐dose paclitaxel with systemic SOX is an effective and tolerable first‐line treatment for patients with peritoneal metastatic gastric cancer and patients with sPCI≤12 scores might be recommended crowd for this regimen as conversion therapy.

## Graphical abstract

Systemic SOX and intraperitoneal high‐dose paclitaxel is an effective first‐line treatment for patients with peritoneal metastatic gastric cancer and patients with sPCI≤12 scores might be recommended crowd for this regimen as conversion therapy.

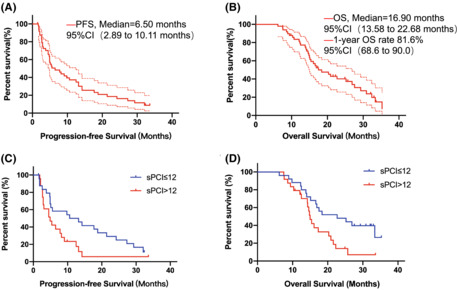

## 1 INTRODUCTION

Gastric cancer (GC) is the fifth most frequently diagnosed cancer and the third leading cause of cancer‐related deaths worldwide with an extremely high incidence in East Asian countries, especially in China and Japan.^1^ Peritoneal dissemination is life‐threatening for GC patients, including synchronous and metachronous metastasis. Further research to identify more effective strategies is crucial for improving treatment outcomes. Systemic chemotherapy is the standard treatment for advanced GC, and intraperitoneal perfusion chemotherapeutics effectively control peritoneal metastasis.^2^, ^3^

Fluorouracil/platinum‐based doublets or triplets systemic chemotherapy is the standard first‐line treatment for advanced GC, as recommended by the West and East clinical guidelines.^4^, ^5^, ^6^ In Asia, S‐1, an oral fluoropyrimidine, has been proven to be superior to 5‐FU and is more commonly used in palliative settings.^7^, ^8^ ACTS‐GC study verified that S‐1 as adjuvant chemotherapy could decrease peritoneal metastasis in patients with resected GC.^9^ REAL2 study showed that oxaliplatin was not inferior to cisplatin but with lower and more tolerated toxicities,^10^ and then, oxaliplatin has been an alternation in palliative chemotherapy. Therefore, SOX is recommended as one of the standard regimens for advanced GC according to the Japanese and Chinese guidelines.^4^, ^5^

At present, anticancer drugs are mainly administered intravenously; most of them cannot cross the peritoneum‐serum barrier and reach the disseminated nodule efficiently. Over the past few decades, intraperitoneal administration of anticancer drugs, such as cisplatin, paclitaxel, mitomycin, and docetaxel, has been proven to be effective for peritoneal metastasis.^11^, ^12^, ^13^ In all of these drugs, paclitaxel is slowly absorbed when applied in intraperitoneal therapy because of its large molecular weight, fat solubility, high AUC peritoneum/plasma and longest penetration distance.^14^, ^15^ Therefore, paclitaxel is considered an ideal chemotherapeutic drug for intraperitoneal administration based on pharmacokinetics. In the phase II clinical trial, intraperitoneal and intravenous paclitaxel plus S‐1 were verified to be safe and effective for treating GC with peritoneal metastasis.^16^ A randomized phase III study called PHOENIX‐GC compared intraperitoneal and intravenous paclitaxel plus S‐1 (IP arm) with cisplatin plus S‐1 (SP arm). The results did not show statistical superiority of the IP arm in the ITT population, but the subgroup data showed that patients with undifferentiated histologic type or moderate ascites could benefit from the IP regimen.^17^ In addition to the reasons given by the authors, the whole regimen in the PHOENIX‐GC study was doublets chemotherapy, and the dosage of paclitaxel administered by intraperitoneal infusion was 20 mg/m^2^, which might mean that the intensity of either systemic or intraperitoneal chemotherapy was relatively weak. V325 and FLOT studies have demonstrated that the triplet regimen is superior to doublets chemotherapy in PFS and OS.^18^, ^19^ The combination of intravenous plus intraperitoneal administration of paclitaxel also led to a dosage restriction applied by intraperitoneal infusion. It is well known that the concentration of chemotherapeutics in the peritoneal cavity is one of the key factors controlling peritoneal metastasis. Any strategy to improve the concentration of chemotherapeutics in the peritoneal cavity deserves our efforts on the premise of safety and controllable toxicity.

Hence, we developed a new intensive regimen that added intraperitoneal high‐dose paclitaxel to systemic chemotherapy SOX with a modified dosage to decrease the potential risk of severe toxicity and conducted a phase II study to evaluate the efficacy and safety of the triplets combination in GC patients with synchronous peritoneal metastasis.

## 2 METHODS

### 2.1 Study design

This was a single‐center, prospective, single‐arm phase II study of intravenous oxaliplatin with oral S‐1 plus intraperitoneal paclitaxel in patients with synchronous peritoneal metastatic GC. The protocol was approved by the Ethics Committee of West China Hospital, Sichuan University, and informed consent was obtained from all patients, and the study was carried out in accordance with the Helsinki declaration and its later amendments. This trial was registered with the Chinese Clinical Trial Registry (ChiCTR1900025984).

### 2.2 Study patients

Patients diagnosed with primary gastric cancer by histopathology or cytology confirmed synchronous peritoneal metastasis by biopsy of peritoneal nodules and exfoliative cytology. Other inclusion criteria were as follows: Eastern Cooperative Oncology Group (ECOG) performance status 0–2 scores, age 18 to 80, measurable or assessable lesions according to the criteria of RECIST 1.1 criteria, without other metastatic organs, and adequate organ function suitable for systemic and intraperitoneal chemotherapy. All enrolled patients underwent a multidisciplinary team (MDT) discussion. The major exclusion criteria were anaphylaxis to the drugs, massive ascites, other metastases except for peritoneal, peritoneal infection, gastrointestinal hemorrhage or obstruction, uncontrolled severe complication and underlying disease.

### 2.3 Study procedures

The primary endpoint was 1‐year overall survival rate, and the second endpoints were PFS, OS, ORR, DCR, and adverse events. Physical examination, abdomen and chest CT were conducted to define the stage of disease, diagnose peritoneal metastasis and evaluate the efficacy every 3 cycles. Before each course of treatment, medical history, physical examination and laboratory examinations (blood cell count, liver and renal function tests, and serum tumor‐related markers including CEA, CA153, and CA125) were performed. Complete blood cell count and liver and renal function (including urinalysis) tests were performed weekly during the treatment. Laparoscopic exploration and gastroendoscopy were also performed when necessary. Tumor responses were assessed according to the Response Evaluation Criteria in Solid Tumor (RECIST) guidelines version 1.1, every 9 weeks. The number of malignant ascites and peritoneal cytology were also considered to evaluate the antitumor effects of the treatment on peritoneal metastasis. The incidence and severity of treatment toxicities were evaluated according to the National Cancer Institute‐Common Terminology Criteria for Adverse Events version 4.0 (CTCAE v4.0).

### 2.4 Treatments

In our study, paclitaxel was administered intraperitoneally on day 1 with an initial dose of 80 mg/m^2^, together with another 1000 ml saline solution. Oxaliplatin was administered intravenously at a modified dose of 100 mg/m^2^ on day 1. S‐1 was administered orally at a dose of 80 mg/m^2^ for 14 days followed by a 7‐day rest. A catheter was implanted in the subcutaneous space of the lower abdomen into the pelvic cavity under ultrasound guidance. The catheter was implanted at the start of every cycle and extubated after infusion. To minimize hypersensitivity reactions to paclitaxel, patients were premedicated with dexamethasone (20 mg), ranitidine (20 mg), and diphenhydramine (20 mg) 30 min before paclitaxel administration. Intraperitoneal and combined systemic chemotherapy were generally administered on an inpatient basis for a maximum of 8 cycles followed by maintenance S‐1 therapy (40 mg/m^2^ twice daily) until transferred to CY0, disease progression, unacceptable toxicity, patient refusal or undergoing radical surgery. The recommended second‐line therapy in our study was doublets chemotherapy including docetaxel, cisplatin, and irinotecan with or without intraperitoneal cisplatin, which was consistent with Western and Eastern guidelines.^4^, ^5^

### 2.5 Statistical analysis

The primary endpoint was 1‐year survival rate, and recent studies in advanced gastric cancer with peritoneal metastasis showed that the 1‐year survival rate was approximately 70%. The sample size was calculated based on the hypothesis that the experimental regimen's 1‐year survival rate could improve by 15%. A one‐sided, one‐sample log‐rank test calculated from a sample of 43 subjects achieved 90.5% power at a 0.025 significance level to detect a 1‐year survival rate of 85% in the experimental group and the expected number of outcome events was 14 during the study. The loss of follow‐up rate was supposed to be 10%; therefore, a total of 47 patients needed to be enrolled. Survival analysis was performed using the Kaplan–Meier method with a log‐rank test. Univariate and multivariate survival analyses were performed using the Cox proportional hazards regression model to generate a hazard ratio using SPSS (version 23.0, SPSS Institute, USA). Rate calculation and 95% Confidence Interval (CI) were done by the “Hmisc” R package with the Wilson test. In all the statistical tests, P‐values ˂0.05, between each group, were deemed to show a statistically significant difference.

## 3 RESULTS

### 3.1 Study population

**FIGURE 1:**
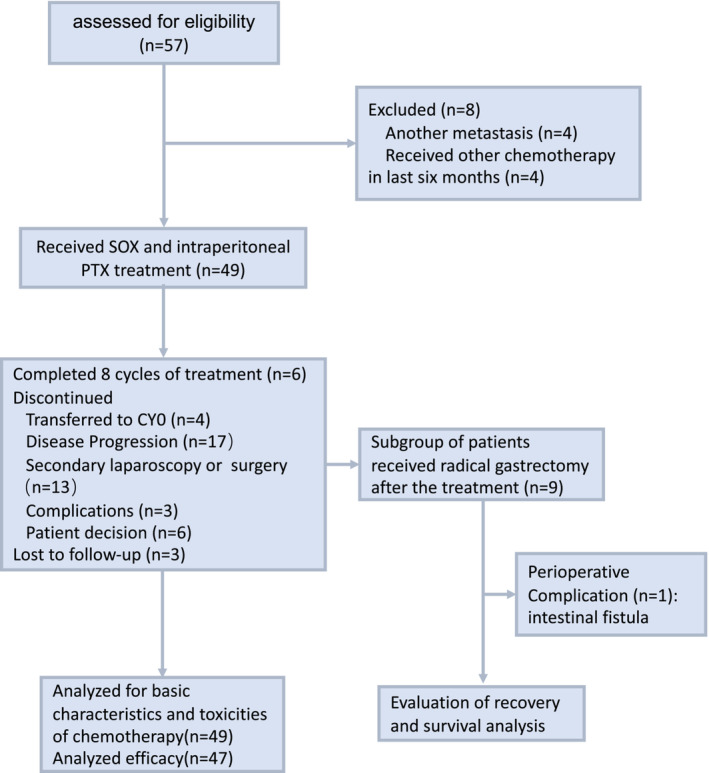
Trail profile at the cutoff date for analysis. Fifty‐seven patients were assessed and 49 patients were enrolled. Forty‐seven patients were included in the survival analysis and two patients were excluded because they did not receive an efficacy evaluation.

Between January 2017 and May 2019, 57 patients were recruited. 8 patients were excluded and 49 eligible patients were finally enrolled in the study (Figure 1). The median age was 55 years (range, 30–78 years), and 89.8% of patients had an ECOG performance status of 0 or 1. The patient characteristics are listed in Table 1. Of the 49 patients, 6 received palliative gastrectomy, 43 underwent laparoscopic exploration and 63.3% of patients were undifferentiated and low differentiated adenocarcinoma. Peritoneal cancer index (PCI) was calculated according to laparoscopic or surgical records by the tumor in the abdominopelvic regions and lesion size score,^20^, ^21^ and the median was 12 (IQR: 4–15) (Table 1).

**TABLE 1:** Characteristics of patients (*n* = 49)

Characteristic	No. of patients	%
Gender		
Male	28	57.1
Female	21	42.9
Age		
Median	55	
Range	30–78	
ECOG ps		
0	25	51.0
1	19	38.8
2	5	10.2
Diagnostic methods of peritoneal metastasis		
Single positive of biopsy of peritoneal nodules	27	55.1
Single positive of exfoliative cytology	14	28.6
Double positive of biopsy and exfoliative cytology	8	16.3
Previous interventions		
Palliative gastrectomy	6	12.2
Laparoscopic exploration	43	87.8
Lauren's type		
Intestinal	21	42.9
Diffuse	28	57.1
Histology		
Un‐ and low differentiated adenocarcinoma	31	63.3
Moderate and high differentiated adenocarcinoma	18	36.7
Amount of ascites^*^		
None	14	28.6
Small	29	59.2
Moderate	6	12.2
sPCI scores		
Median	12	
IQR	4–15	
No. of cycles		
Median	3	
IQR	3–6	

Abbreviations: ECOG PS, Eastern Cooperative Oncology Group performance status; IQR, interquartile range; PCI, peritoneal cancer index; sPCI, PCI was calculated using laparoscopy or surgery record.

^*^ Ascites were evaluated by computed tomography or laparoscopy: small indicates ascites within the pelvic cavity; moderate indicates ascites beyond the pelvic cavity.

### 3.2 Treatment outcomes

**FIGURE 2:**
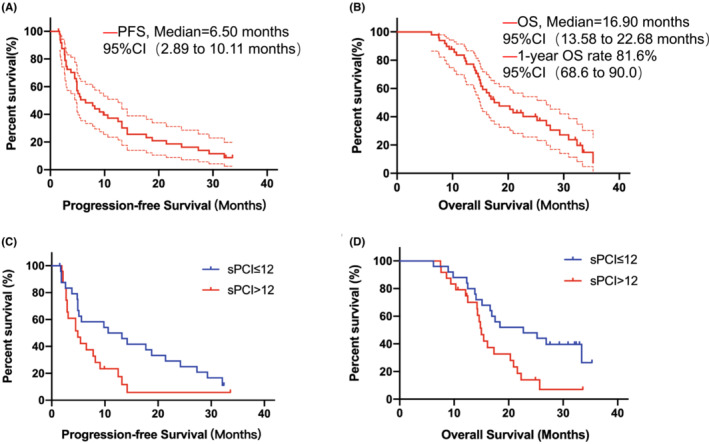
Survival analysis. (A) Progression‐free survival and (B) Overall survival analysis of all patients; (C and D) Subgroup analysis of sPCI, comparison of progression‐free survival and overall survival between sPCI ≤12 and. sPCI >12; *The area between the red dotted lines indicates the 95% confidence interval.** Short, vertical lines on the survival curve indicate censored data.

Up to December 31, 2020, the median follow‐up time for censored patients was 32.4 months (95% CI, 31.05–33.75). The median PFS was 6.50 months (95% CI, 2.89–10.11) and the median OS was 16.9 months (95% CI, 13.58–20.22) (Figure 2). Forty‐seven patients received at least one efficacy evaluation, and the 1‐year overall survival rate was 81.6% (95% CI, 68.6–90.0). ORR was 55.3% (95% CI, 41.3–68.6) and DCR was 76.6% (95% CI, 62.8–86.4) (Table 2). Thirteen patients had a secondary operation following the decision of MDT, during which nine of them underwent radical surgery and were R0, D2 radical gastrectomy. For the nine patients, the median PFS was 27.3 months (95% CI, 24.96–29.64), and the median OS was 33.4 months (95% CI, 30.74–36.06).

**TABLE 2:** Efficacy evaluation (*n* = 47)

	No. of patients	% (95% CI)
Overall efficacy evaluation (*n* = 47)		
Complete response	0	0
Partial response	21	51.2 (36.5–65.8)
Stable disease	15	24.4 (13.8–39.3)
Progression disease	11	24.4 (13.8–39.3)
Secondary laparoscopy	13 (*n* = 41)	31.7 (19.0–47.0)
Radical surgery	9 (*n* = 41)	22.0 (12.0–36.7)
ORR	21 (*n* = 41)	51.2 (36.5–65.8)
DCR	36	76.6 (62.8–86.4)

Abbreviations: DCR, Disease Control Rate; ORR, Overall Response Rate.

Further subgroup analysis of baseline characteristics and treatment for survival was conducted, and the results showed that pre‐treatment sPCI was a significant prognostic factor for PFS and OS (Table 3 and Figure 2). The median PFS was 10.6 months (95% CI, 0.3–20.9 months) in sPCI ≤12 subgroup and 4.9 months (95% CI, 3.6–6.2 months) in sPCI >12 subgroup (HR, 1.89; 95% CI, 0.96–3.75; *p* = 0.067; in Table 3); the median OS was 22.7 months (95% CI, 10.1–32.2 months) in sPCI scores ≤12 subgroup and 15.0 months (95% CI, 13.8 to 16.2 months) in sPCI >12 subgroup (HR, 2.24; 95% CI, 1.13–4.43; *p* = 0.021; in Table 3).

**TABLE 3:** Subgroup analysis of survival

Factor	Univariable		Multivariable	
	HR (95% CI)	*p* value	HR (95% CI)	*p* value
Progression‐free survival				
Gender (Male vs. Female)	0.79 (0.43–1.47)	0.458	0.98 (0.44–2.22)	0.970
Age (≤55 vs. >55)	1.44 (0.77–2.67)	0.255	1.16 (0.58–2.33)	0.682
ECOG ps (≤1 vs. >1)	1.56 (0.84–2.93)	0.162	1.65 (0.86–3.16)	0.131
Histology (SRCC vs. Ade)	0.96 (0.50–1.83)	0.890	0.78 (0.36–1.70)	0.528
PCI (≤12 vs. >12)	1.86 (0.97–3.56)	0.060	1.89 (0.96–3.75)	0.067
Overall survival				
Gender (Male vs. Female)	0.72 (0.37–1.38)	0.322	0.78 (0.36–1.69)	0.529
Age (≤55 vs. >55)	0.98 (0.52–1.86)	0.958	0.90 (0.46–1.77)	0.769
ECOG ps (≤1 vs. >1)	1.30 (0.68–2.48)	0.432	1.26 (0.64–2.46)	0.500
Histology (SRCC vs. Ade)	1.01 (0.52–1.96)	0.969	0.88 (0.41–1.92)	0.750
PCI (≤12 vs. >12)	2.24 (1.13–4.43)	0.021	2.19 (1.08–4.42)	0.029

Abbreviations: Ade, Adenocarcinoma; SRCC, Signet‐ring cell carcinoma.

### 3.3 Toxicity assessment

The chemotherapy‐related hematological and non‐hematological adverse effects are listed in Table S1. The most common grade 3/4 toxicities were neutropenia (40.8%), anemia (22.4%), leukopenia (18.4%), nausea (14.3%), and vomiting (12.2%) (Table 4). None of the patients had any complications related to intraperitoneal infusion or catheter obstruction. Three patients had complications, including gastric perforation, chronic intestinal obstruction and renal inadequacy. Among the 9 patients who underwent radical gastrectomy, one patient had perioperative complications, including intestinal fistula. Moreover, there were no treatment‐related deaths.

**TABLE 4:** Toxicity assessment (*n* = 49)

Toxicity	No. of patients of different Grade (CTCAE 4.0)				
	1	2	3	4	3/4%
Anemia	30	8	10	1	22.4
Leukopenia	22	18	9	0	18.4
Neutropenia	13	16	16	4	40.8
Thrombocytopenia	40	6	3	0	6.1
Liver Function					
Bilirubin	47	0	0	0	0
ALT	47	0	0	0	0
AST	47	0	0	0	0
Anorexia	28	17	4	0	8.2
Nausea	21	21	7	0	14.3
Vomiting	26	19	4	0	8.2
Abdominal pain	30	15	2	2	8.2
Diarrhea	35	8	6	0	12.2
Constipation	46	3	0	0	0
Peripheral sensory neuropathy	43	5	1	0	2.0
Alopecia	40	6	3	0	6.1
Rash maculopapular	46	3	0	0	0
Fatigue	28	18	3	0	6.1

Abbreviations: ALT, alanine transaminase; AST, glutamic‐oxaloacetic transaminase; CTCAE, Common Terminology Criteria for Adverse Events.

### 3.4 Discussion

GC with peritoneal dissemination has poor prognosis and the median overall survival has been reported to be 6–10 months.^3^ Intraperitoneal infusion of chemotherapeutics, especially taxanes, could prolong the overall survival because it could lead to a very high concentration of drugs in the peritoneal cavity, which means lower systemic toxicities. Studies show that responses to taxanes seem to be dose‐dependent.^22^, ^23^ Hence, increasing doses of paclitaxel by intraperitoneal administration might be an effective strategy for peritoneal metastasis. This study evaluated the efficacy and safety of a triplet regimen consisting of intraperitoneal high‐dose paclitaxel combined with intravenous oxaliplatin and oral S‐1 in 49 GC patients with synchronous peritoneal metastasis. The median survival of PFS and OS were 6.5 months and 16.9 months and the 1‐year OS rate was 81.6%. Systemic and abdominal toxicities are mild and manageable.

To our knowledge, this is the first report of the combination of intraperitoneal high‐dose paclitaxel with modified SOX in advanced gastric cancer with peritoneal metastasis. The 1‐year OS rate reached 81.6%, which is a little higher than that reported in previous studies.^16^, ^17^ The reason might be the dose of paclitaxel was 80 mg/m^2^ in this study, which allowed an extremely high concentration of paclitaxel in the peritoneal cavity compared with previous ones,^16^, ^17^ in which paclitaxel was applied weekly two to three times in a cycle with a low dosage. And then, this regimen was composed of paclitaxel, oxaliplatin and S‐1, while the previous PHOENIX‐GC trial regimen was consisted of paclitaxel and S‐1. Platinum drugs such as cisplatin and oxaliplatin are recommended for gastric cancer treatment, and triplet regimens have been verified to be more effective in FLOT and V325 studies.^18^, ^19^ However, the median OS of patients in our study was only 16.9 months, approximately equal to previous studies. Further analysis showed that the reasons might be the lower second‐line treatment rate and higher PCI scores of patients. The number of patients who received second‐line chemotherapy only reached 43% (18/38). The median sPCI was 12 points (IQR: 4–15), which was higher than that of patients in the PHOENIX‐GC trial. The extent of peritoneal metastasis is an important factor that has a great influence on patient survival.^24^

For further research, we performed subgroup analysis of basic characteristics. The results showed that sPCI was a good index for a favorable prognosis. The sPCI cut‐off value for favorable prognosis has not been unified. Previous studies which focused on intraperitoneal infusion used 10 or 20 as the cut‐off values.^16^, ^25^ In this study, the cut‐off value in the ROC curve was 12.5, and the area under the curve was 0.804. Thus, we divided the patients into two subgroups accordingly (sPCI ≤12 vs. sPCI >12). The median OS for sPCI ≤12 subgroup could reach 22.7 months, much longer than the sPCI >12 subgroup.

Nine patients underwent radical gastrectomy and all of them had less than 12 points of sPCI, which means that 36.0% (9/25) of patients whose sPCI scores were less than 12 points had the opportunity to undergo radical gastrectomy after treatment with this regimen. The median OS of the 9 patients could reach 33.4 months, which means that patients with sPCI ≤12 in synchronous peritoneal metastasis of gastric cancer might be recommended crowd for this regimen as conversion therapy. Over the past decades, cytoreductive surgery (CRS) plus hyperthermic intraperitoneal chemotherapy (HIPEC) therapy has been developed, and its application in gastric cancer with peritoneal metastasis has also become more widespread.^26^ A retrospective study conducted by Li et al.^27^ showed that the median OS of patients could reach 30.3 months after receiving CRS plus HIPEC in the CC0 subgroup. This regimen had a better OS in selected patients. However, the incidence of serious adverse events in CRS plus HIPEC was higher than that in this regimen. The statistics also showed that CRS plus HIPEC treatment‐related deaths occurred at a rate of 0%–11%.^26^ What's more, many hospitals in China do not have HIPEC equipment to put CRS plus HIPEC into effect and this regimen could be an alternative for patients, especially patients with PCI ≤12 subgroups.

Since we increased the dose of paclitaxel to 80 mg/m^2^, toxicity is one of the most important factors for applying the regimen. The results showed that toxicities, both hematologic and non‐hematologic, were controllable and acceptable in this study. Considering the potential toxicity of triplet regimens, the dose of intravenous oxaliplatin was decreased from 130 mg/m^2^ to 100 mg/m^2^.^5^, ^24^ And paclitaxel was administered only by intraperitoneal infusion, which means a lower concentration in circulation. In this study, none of the patients had any problems related to intraperitoneal catheter‐like obstruction and infection. This is because the catheter was placed at the start of every cycle and extubated after infusion. Three patients had complications, including gastric perforation, chronic intestinal obstruction and renal inadequacy. Basic information on these patients can provide reasonable explanations including stomach ulcers and high scores of sPCI. The patient with gastric perforation had relatively risky ulcer of the stomach before treatment and he only received 1 cycle treatment before the stomach perforation occurred. The patient with chronic intestinal obstruction had 23 points of sPCI and the progression‐free survival of the patient was only 2.7 months, peritoneal metastasis and primary cancer might be the reasons for chronic intestinal obstruction. The treatment of patients with adverse events was consistent with clinical guidelines and details were reported to relative departments in time.

There are some limitations to our study. First, it was a single‐arm phase II study, which made it impossible to compare 80 mg/m^2^ of paclitaxel once a cycle with 20 mg/m^2^ twice a cycle directly, and patients were enrolled only from our hospital, all of whom were Chinese. The efficacy evaluation of patients was performed by researchers of this study but not an independent evaluation committee. We do not have pre‐clinical pharmacokinetic research on high‐dose paclitaxel administered by intraperitoneal infusion, which is crucial for further clinical administration.

In conclusion, the combination of intraperitoneal high‐dose paclitaxel and modified SOX is a safe therapy for advanced GC with peritoneal metastasis. Patients with sPCI ≤12 in synchronous peritoneal metastasis of GC should be recommended as conversion chemotherapy for this regimen. Compared with previous studies, it is more convenient and possibly more effective in some patients, but further clinical trials are necessary for a larger group of patients.

## AUTHOR CONTRIBUTIONS

Study concept and design: Meng Qiu, Jiankun Hu. Data acquisition: Li Tu, Ni Lu. Quality control of data: Wei‐Han Zhang. Data analysis and interpretation: Li Tu, Meng Qiu. Statistical analysis: Li Tu, Zi‐Han Xu, Kun Yang. Manuscript preparation: Li Tu, Wei‐Han Zhang. Manuscript editing: Meng Qiu. Manuscript review: Meng Qiu, Jiankun Hu. Administrative, technical, or material support: Hong‐Feng Gou, Qing Zhu, Ming Liu, Yu Yang.

## FUNDING INFORMATION

This work was supported by the National Key Development Plan for Precision Medicine Research (2017YFC0910004).

## CONFLICT OF INTEREST

No authors report any conflict of interest.

## ETHICS

All procedures followed were in accordance with the Declaration of Helsinki and the study was approved by the Institutional Review Board of our center. All patients provided written informed consent before enrollment.

## Data Availability

The data that support the findings of this study are available from the corresponding author upon reasonable request.
